# The German Hospital, Dalston

**Published:** 1910-02-19

**Authors:** 


### EDITOR'S LETTER-BOX.

### THE GERMAN HOSPITAL, DALSTON.

*To the Editor of* THE HOSPITAL.

SIR,—My attention has been drawn to a paragraph in the last edition of THE HOSPITAL, in which reference is made to the overcrowded state of the wards of the German Hospital.

I take the liberty of enclosing a statement giving the areas of all the wards occupied by patients, from which it will be seen that, in every case, the cubical air space per bed is fully 20 per cent. above the generally recognised standard for ordinary patients, namely, 1,200 cubic feet for adults, and 600 cubic feet for children.

I trust these figures will convince you that the evils referred to by your informant are practically non-existent, and that consequently you will be good enough to cause a paragraph to this effect to appear in the next issue of your journal.

I am, sir, yours faithfully,

BARON SCHRÖDER.
Treasurer.

German Hospital, Dalston, London, N.E.,
February 11, 1910.

*Areas of Wards.*

| | | | | | |
|---|---|---|---|---|---|
| Children's Ward (21 Cots)— 39′ 8″×24′ 9″×15′ 11″ high | 15,625 | c. ft. | 744 | c. ft. | per cot |
| Accident Ward (15 beds)— 58′ 3″×9′ 10″×15′ 1″ high | 21,713 | ,, | | | |
| Less cross wall | 280 | | | | |
| | 21,433 | ,, | 1,430 | ,, | per bed |
| Ward 4 (20 Beds)— 79′ 10″×24′ 10″×15′ 1″ high | 29,922 | ,, | | | |
| Less cross wall | 280 | | | | |
| | 29,642 | ,, | 1,432 | ,, | ,, |
| Ward 6 (10 Beds)— 39′ 0″×24″ 10″×15′ 0″ high | 14,537 | ,, | 1,453 | ,, | ,, |
| Ward 5 (10 Beds)— 39′ 8″×24″ 10″×15′ 0″ high | 14,783 | ,, | 1,478 | ,, | ,, |
| Ward 7 (20 Beds)— 79′ 10″×25′ 1″×15′ 0″ high | 30,040 | ,, | | | |
| Less 4′ 6″×5′ 3″×79′ 10″ | 1,885 | ,, | | | |
| | 28,155 | ,, | 1,407 | ,, | ,, |
| Ward 8 (20 Beds)— 80′ 0″×25′ 0″×15′ 1″ high | 30,160 | ,, | | | |
| Less for sloping ceiling, 4′ 6″×5′ 3″×80′ 0″ | 1,889 | ,, | | | |
| | 28,281 | ,, | 1,414 | ,, | ,, |

The dimensions of the rooms adjoining wards, and of the rooms for private patients, are not included, but these are also fully up to standard requirements.

[We have pleasure in publishing Baron Schröder's letter. It is, however, the question of floor space which is, after all, the first thing to be considered in planning a hospital ward. By way of illustration, we may mention a case which occurred in India where instructions were given to erect a hospital for a fixed number of beds, with the proviso that to each bed a certain amount of cubic space was to be allotted. When the hospital was built the wards contained the cubic space asked for, but when the bedsteads were sent down it was found that the cubic space had been obtained by making the wards so high that the proper number of beds could only be got in by covering the whole floor with them, so that no space was left to get to the patients. At the German Hospital, as the figures show, the height of the wards is two, or, as we hold, three feet—and in the case of the children's ward nearly four feet—too high. Each ward should contain a minimum floor space of 100 feet. Some authorities on ventilation consider that any excess of height over twelve feet ought to be neglected in calculations affecting the movement of air. None of the wards at the German Hospital are sufficiently large to require, for effective ventilation and appearance, a greater height than twelve feet. The atmosphere of the wards confirms the opinion expressed by Dr. Billings in his book on Ventilation and Warming, that any excess of height above twelve feet must be neglected in calculations affecting the movement of the air. Apart from this the figures given of the size and contents of the children's ward confirm the justice of the criticism, that it is at present crowded, and that the number of cots should be reduced from twenty-one to say sixteen or seventeen as a maximum. In justice to the authorities, we ought to add that the buildings of the German Hospital are old, and have been in use, we believe, for quite forty years. The effective ventilation of hospital wards and their hygienic requirements were imperfectly understood forty years ago.—Editor, The Hospital.]

THE HOSPITAL. February 19, 1910.

